# Case Report: Neonatal PURA syndrome caused by a novel c.463C>G (p.Tyr155Ter) mutation

**DOI:** 10.3389/fped.2026.1811556

**Published:** 2026-03-31

**Authors:** Yongxin Wang

**Affiliations:** Department of Neonatology and Neonatal Intensive Care, Jieyang People's Hospital, Jieyang, China

**Keywords:** feeding difficulties, genetic variant, hypotonia, neonate, neurodevelopmental disorder

## Abstract

PURA syndrome is a rare genetic disease characterized by significant phenotypic variability. This case report presents a 4-day-old female neonate presenting with hypotonia, feeding difficulties, and other symptoms. Following comprehensive clinical examination, including laboratory tests, imaging studies, and genetic analysis, the patient was diagnosed with PURA syndrome. Whole-exome sequencing was performed on blood samples collected from the patient and her parents, revealing a novel *PURA* gene mutation [c.463C>G (p.Tyr155Ter), NM_005859.5], which had not been previously recorded in the literature. This case report aims to expand the known genotype of PURA syndrome and support clinicians in early detection and diagnosis. Early diagnosis facilitates immediate initiation of targeted swallowing function assessment and rehabilitation training, prevents aspiration pneumonia, and guides families in genetic counseling to avoid recurrence risk.

## Introduction

PURA syndrome is an autosomal dominant genetic disorder caused by *PURA* gene variants, primarily manifesting as neurodevelopmental delay, hypotonia, feeding difficulties, and epilepsy (1). The disease was first reported in 2014 (2), with limited cases reported globally to date. The novel *PURA* gene variant [c.463C>G (p.Tyr155Ter), NM_005859.5] identified in our patient has not been previously recorded. Searches of the ClinVar, ClinGen, gnomAD (v3.1.2, accessed October 2024), and PubMed databases confirmed that this variant is novel and unreported. This case contributes to expanding the clinical and genetic understanding of PURA syndrome and highlights the value of genetic testing in accurate diagnosis and early intervention.

## Patient information

The patient was a female infant, 4 days postpartum, admitted due to “feeding difficulties and jaundice for 2 days.” The child was the second pregnancy and second birth, with a gestational age of 39 + 4 weeks, delivered via cesarean section due to “excessive fetal head size.” After birth, the infant was observed in the maternity mother-baby room. The mother was discharged on the second day postpartum, but the infant was brought to our emergency department by family members on the third day of life due to weak sucking and worsening jaundice, and was subsequently admitted to the neonatal ward for treatment.

Both parents were healthy and non-consanguineous. Father's blood type: O RhD(+), mother's blood type: B RhD(+). An older brother was born at 35 weeks via spontaneous vaginal delivery and was diagnosed with trisomy 21 (Down syndrome). Parental age at reproduction: father 34 years, mother 32 years (when giving birth to the proband); father 31 years, mother 29 years (when giving birth to the older brother). Following the sibling's diagnosis, both parents underwent peripheral blood karyotype analysis, which showed normal results with no Robertsonian translocations detected, indicating the sibling's trisomy 21 was a random *de novo* occurrence with no direct genetic association to the *PURA* gene mutation in this patient. The mother underwent regular prenatal checkups; first-trimester NT examination and second-trimester serological screening (Down syndrome screening) were both low-risk, and systematic ultrasound anomaly screening revealed no obvious structural abnormalities.

### Physical examination

Temperature: 36.8 °C, body weight 3.6 kg (P50–75), body length 51 cm (P50), head circumference 35 cm (P50). General response was poor, crying was weak, moderate jaundice throughout the body with scleral icterus. No obvious hypothermia or persistent hiccups were observed. Breathing was slightly rapid, cardiac examination was unremarkable, limb muscle tone was low, limb movements were reduced, alertness was decreased with minimal response to stimulation. No seizures or abnormal movements were observed. Moro reflex, grasp reflex, rooting reflex, and sucking reflex were all diminished.

## Examination results

### Blood tests

Total bilirubin 16.1 mg/dL, indirect bilirubin 14.5 mg/dL, direct bilirubin 1.6 mg/dL; Coombs test negative; G6PD normal (10 U/g Hb).

### Imaging studies

**Figure 1:**
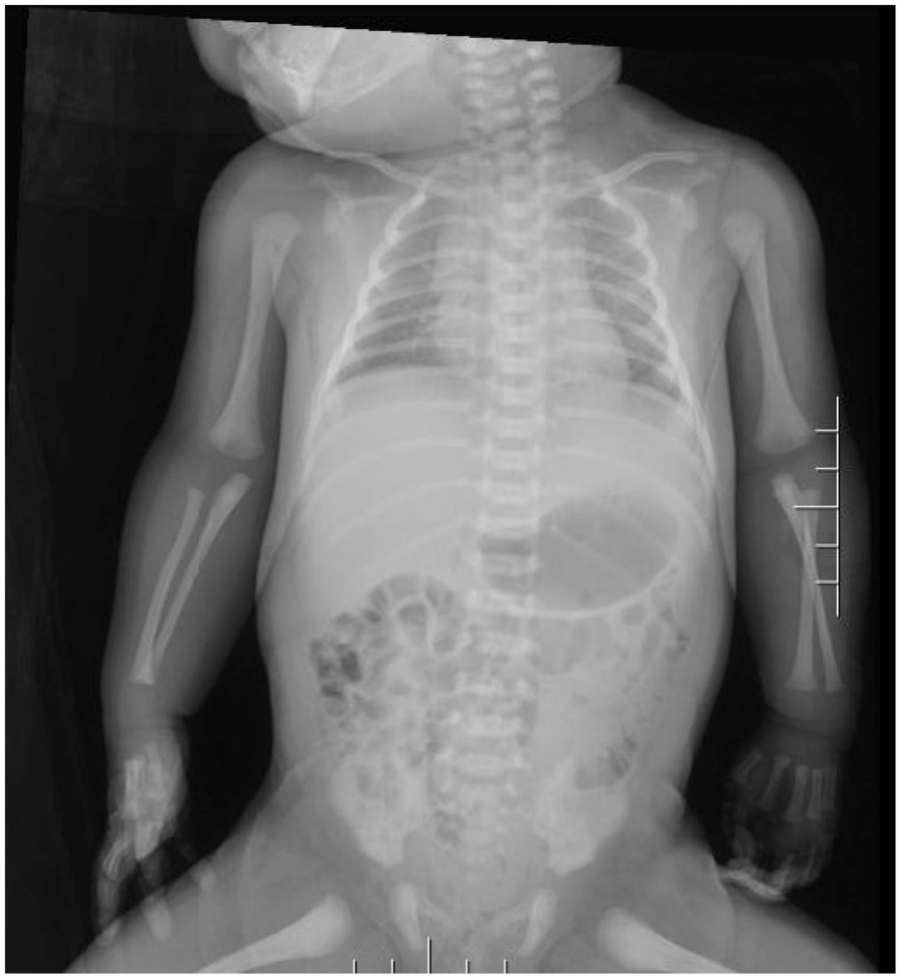
Chest radiograph. Chest radiograph demonstrates increased lung markings, particularly in bilateral lung fields. Cardiac silhouette appears within normal limits for age. No focal consolidation, pleural effusion, or pneumothorax is identified.

**Figure 2:**
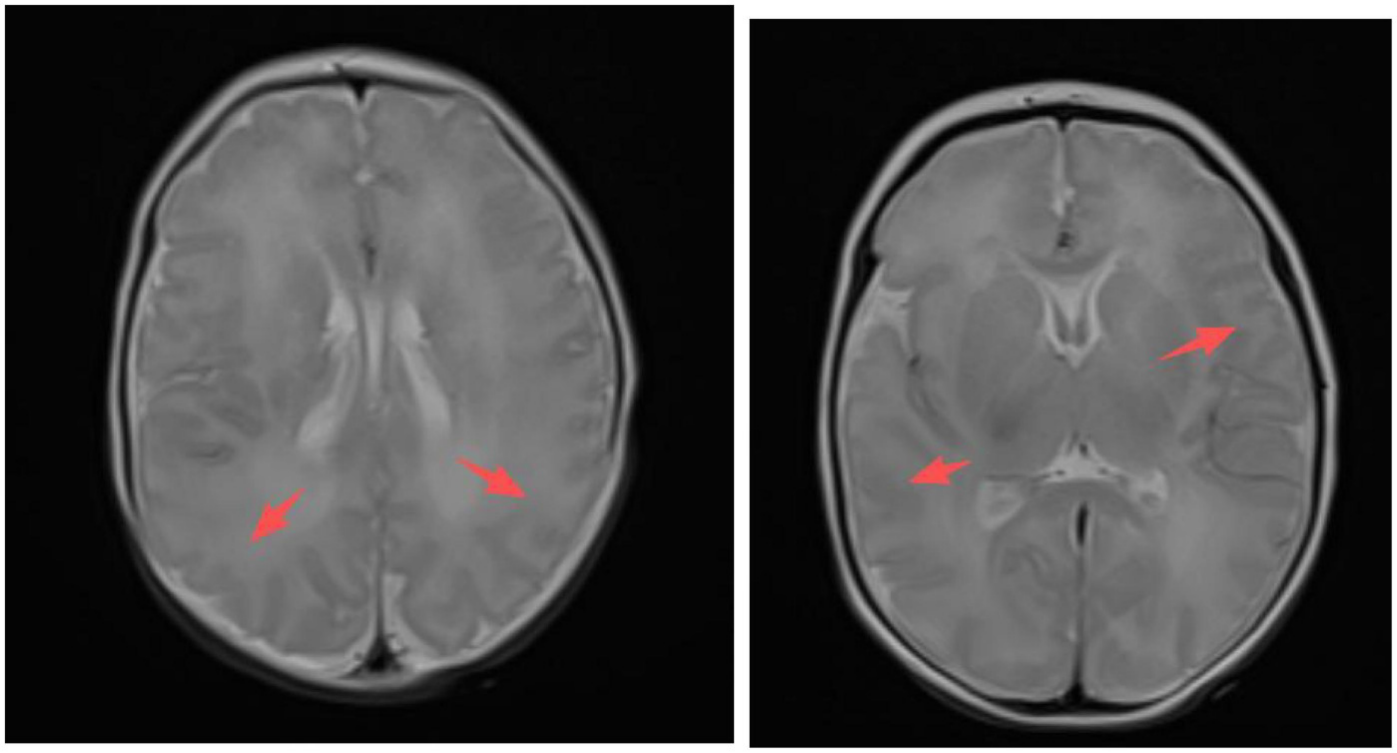
Abnormal brain MRI. Axial T2-weighted image demonstrates slightly swollen gyri with shallow sulci. Poor demarcation between gray and white matter. The normal distinct interface between cortex and white matter is blurred.

- Chest x-ray (see Figure 1) shows mild increased lung markings, particularly in the middle and lower lung fields, with generalized blurring of bilateral markings, but no obvious consolidation. No obvious pneumonia observed on chest radiograph.
- Cardiac ultrasound: Patent ductus arteriosus (1.56 mm, left-to-right shunt)
- Urinary system ultrasound, abdominal organ ultrasound, and head ultrasound were all unremarkable
- Brain MRI (see Figure 2): Poor demarcation between gray and white matter, slightly swollen gyri.
- EEG was not performed because there were no clinical seizures and neurological status was stable

### Genetic testing results returned 27 days after birth (sequencing depth 100x, variant allele frequency 50%)

**Figure 3:**
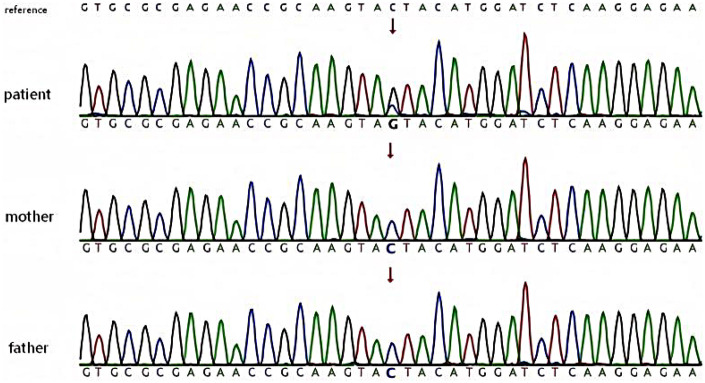
Sanger sequencing verification results. Top: *PURA* gene (NM_005859.5) c.463C>G heterozygous mutation peak chromatogram of the child; Middle: father's wild-type sequence; Bottom: mother's wild-type sequence. Red arrow indicates mutation site.

A heterozygous variant c.463C>G (p.Tyr155Ter) was found in exon 1 of the child's *PURA* gene. Parents had no variant at this locus. This variant is a novel nonsense variant and classified as a pathogenic variant according to ACMG guidelines. Based on the child's clinical characteristics and genetic test results, PURA syndrome was diagnosed (see Figure 3).

### ACMG classification criteria

- **PVS1 (Very Strong)**: This variant is a nonsense mutation causing premature termination of protein translation (p.Tyr155Ter). Given that the *PURA* gene is highly sensitive to haploinsufficiency and this truncating variant is located early in the coding region, it is predicted to trigger nonsense-mediated mRNA decay (NMD), thus assigning the highest strength evidence of PVS1.
- **PS2 (Strong)**: This variant is a *de novo* variant confirmed by family segregation analysis.
- **PM2 (Moderate)**: This variant was not found in reference population databases including 1000 Genomes (1000G), Shenzhou Genome Database, Exome Aggregation Consortium (ExAC), and genome aggregation database (gnomAD); search date: October 1, 2024.

## Treatment interventions

### First hospitalization (postnatal day 4)

Admitted to neonatology ward with symptoms of jaundice and respiratory distress. Management included nasal cannula oxygen therapy, phototherapy and intravenous fluids. For feeding difficulties, gastric tube feeding was initiated and swallowing therapy was provided by a rehabilitation specialist to assess oral motor function, suck-swallow-breathe coordination and aspiration risk. The patient was discharged on postnatal day 14, and continued outpatient rehabilitation therapy 10 times. After therapy, the patient was able to feed independently with 80 mL per feeding.

### Second hospitalization at 42 days of life

The child was hospitalized in the pediatric department of our hospital for pulmonary infection, with elevated inflammatory markers and positive SARS-CoV-2 test. Treatment included nasal cannula oxygen, anti-infection therapy, nebulization, and expectorant therapy. During hospitalization, sucking function weakened, requiring nasogastric tube feeding. The child had weak coughing ability and poor sputum clearance, with deteriorating pulmonary function requiring invasive respiratory support. The family discharged the child automatically after 4 days of hospitalization.

The child died at 47 days of life.

## Discussion

PURA syndrome is a genetic disorder caused by variants in the *PURA* gene. In 2014, Lalani et al. first identified the *PURA* gene as the key gene in the critical deleted region in patients with 5q31.3 microdeletion syndrome. The encoded Pur-α protein is a highly conserved, ubiquitously expressed multifunctional nucleocytoplasmic shuttling protein (3). It has diverse biological functions including involvement in fundamental molecular processes such as DNA replication, transcriptional regulation and mRNA transport (4, 5), and plays critical roles in neurodevelopment (6), suggesting that pathogenic variants in *PURA* gene may affect nervous system development.The *PURA* gene c.463C>G (p.Tyr155Ter) nonsense variant identified in this case is novel, causing premature termination of protein synthesis and predicted to trigger nonsense-mediated mRNA decay (NMD), resulting in complete loss of functional PURA protein. This supports assigning PVS1 (Very Strong) pathogenicity evidence, as NMD-mediated protein loss of function is the well-established mechanism of PURA syndrome pathogenesis. To date, no genotype-phenotype correlations have been identified, likely due to limited sample size (7–9).

PURA syndrome primarily presents with neonatal hypotonia, feeding difficulties, lethargy, respiratory distress, developmental delay, epilepsy and motor dysfunction (1). This patient presented with feeding difficulties and hypotonia in the neonatal period, consistent with typical clinical manifestations of PURA syndrome. Brain MRI showed poor demarcation between gray and white matter and slightly swollen gyri. According to published case series, common neuroimaging findings in PURA syndrome may include delayed or hypomyelination, nonspecific white matter abnormalities, parenchymal atrophy, corpus callosum dysgenesis, or cerebellar vermis hypoplasia (8–10). The poor gray-white matter demarcation in this case may reflect early myelination abnormalities, consistent with white matter abnormalities reported in literature. However, typical findings such as brain atrophy or corpus callosum dysgenesis were not observed, suggesting heterogeneity in neuroimaging phenotypes of PURA syndrome, warranting larger sample studies to further characterize this spectrum. Additionally, the patient exhibited non-specific symptoms including jaundice and patent ductus arteriosus, which may be consistent with multi-system involvement in PURA syndrome. However, these are common neonatal findings, and definitive causal relationships require further investigation. Previous studies have documented diverse clinical manifestations in PURA syndrome, including cardiovascular and liver abnormalities. A recent systematic review of PURA cases identified common features among Asian cases such as hypotonia (95.5%), feeding difficulties (82.9%) and need for respiratory support (63.6%). Table 1 presents a comparison between this case and the Asian case series (10).

**Table 1:** PURA case comparison

Feature	This Case	Asian Patient (*n* = 44)
Hypotonia	Yes	95.2%
Feeding difficulties	Yes	82.9%
Respiratory support	No	63.6%
Seizures/EEG abnormalities	No	10.5%
Normal MRI	No	27%
Early mortality	Yes	6.8%

Differential diagnoses include other neurodevelopmental disorders such as Prader-Willi syndrome, Angelman syndrome, and spinal muscular atrophy, as well as non-genetic causes such as hypoxic-ischemic encephalopathy and metabolic disorders. Genetic testing is crucial for definitive diagnosis.

The clinical deterioration observed during readmission occurred in the context of SARS-CoV-2 positive pulmonary infection. It is important to distinguish infection-related clinical decline from baseline PURA phenotype. While baseline PURA phenotype is characterized by hypotonia, feeding difficulties and neurodevelopmental delay, acute decompensation may be exacerbated by viral infection, potentially leading to respiratory failure and cardiovascular instability.

There is currently no specific treatment for PURA syndrome. Early diagnosis enables early intervention including physical therapy, occupational therapy and feeding support to improve developmental outcomes. For patients with feeding difficulties, regular follow-up should monitor for respiratory infections as these patients may be at increased risk for aspiration-related complications due to impaired swallowing function. This patient showed improvement after symptomatic treatment and rehabilitation, but still exhibited developmental delay. Early prevention measures include genetic counseling for the family, as this variant is *de novo* with low recurrence risk.

Clinicians should enhance awareness of PURA syndrome. For neonates presenting with hypotonia and feeding difficulties, *PURA* gene variants should be considered, and genetic testing should be performed early to confirm diagnosis. Management of this condition continues to pose challenges, requiring further research to explore effective therapeutic approaches.

## Data Availability

The original contributions presented in the study are included in the article/supplementary material, further inquiries can be directed to the corresponding author/s.
