# Supplementary material for: Case Report: Neonatal PURA syndrome caused by a novel c.463C>G (p.Tyr155Ter) mutation
Source: Front Pediatr. 2026 Mar 31;14:1811556. doi: 10.3389/fped.2026.1811556 (PMC13076274; doi:10.3389/fped.2026.1811556)
Supplement: Supplementary file 1 [file Supplementaryfile1.docx]

# CARE Checklist for Frontiers in Pediatrics Submission

## 1. Title

• ✅ **Case Report: A Neonatal Case of PURA Syndrome Caused by a Novel c.465C>G (p.Y155) De Novo Mutation**

## 2. Key Words

• ✅ PURA syndrome, neonatal hypotonia, de novo mutation, case report

## 3. Abstract

### 3a. Introduction

• ✅ First reported in 2014, PURA syndrome is a rare neurodevelopmental disorder caused by mutations in the PURA gene. This case describes a novel c.465C>G (p.Y155) de novo mutation in a neonate, expanding the known genetic spectrum of the disease.

### 3b. Main symptoms and clinical findings

• ✅ A 4-day-old female neonate presented with feeding difficulties, hypotonia, and jaundice. Physical examination revealed reduced primitive reflexes and poor sucking ability.

### 3c. Diagnoses, interventions, and outcomes

• ✅ Genetic testing confirmed a heterozygous c.465C>G mutation in the PURA gene. Symptomatic treatment including phototherapy, nasogastric feeding, and rehabilitation was provided. The patient showed partial improvement but required ongoing care.

### 3d. Conclusion

• ✅ This novel de novo mutation expands the genetic landscape of PURA syndrome and highlights the importance of early genetic testing in neonates with hypotonia and feeding difficulties.

## 4. Introduction

• ✅ PURA syndrome is a rare autosomal dominant disorder characterized by neurodevelopmental delay, hypotonia, and epilepsy. This case reports a previously unrecorded c.465C>G (p.Y155) mutation in a neonate, contributing to the growing body of knowledge about this condition.

## 5. Patient Information

### 5a. De-identified patient information

• ✅ 4-day-old female neonate, born via cesarean section at 39+4 weeks gestation, birth weight 3.6 kg.

### 5b. Primary concerns and symptoms

• ✅ Feeding difficulties, jaundice, and hypotonia.

### 5c. Medical, family, and psycho-social history

• ✅ Non-consanguineous parents, older brother with trisomy 21. No family history of genetic disorders.

### 5d. Relevant past interventions with outcomes

• ✅ Initial supportive care in the neonatal unit, including phototherapy for jaundice and nasogastric feeding.

## 6. Clinical Findings

• ✅ Physical examination revealed hypotonia, reduced primitive reflexes, and poor sucking ability. Serum bilirubin was 275.9 μmol/L. Cranial MRI showed indistinct gray-white matter differentiation.

## 7. Timeline

| **Timepoint** | **Event** |
| --- | --- |
| Birth | Cesarean section for large fetal head |
| 2 days old | Onset of feeding difficulties and jaundice |
| 4 days old | Hospital admission, genetic testing initiated |
| 14 days old | Discharge with nasogastric feeding |
| 42 days old | Readmission for pneumonia and SARS-CoV-2 infection |

## 8. Diagnostic Assessment

### 8a. Diagnostic testing

• ✅ Whole-exome sequencing, serum bilirubin measurement, cranial MRI, echocardiography.

### 8b. Diagnostic challenges

• ✅ Rare disease presentation, initial non-specific symptoms.

### 8c. Diagnosis

• ✅ PURA syndrome due to c.465C>G (p.Y155) de novo mutation. Differential diagnoses included hypoxic-ischemic encephalopathy and metabolic disorders.

### 8d. Prognosis

• ✅ Guarded prognosis with expected developmental delay and ongoing medical needs.

## 9. Therapeutic Intervention

### 9a. Types of intervention

• ✅ Phototherapy for jaundice, nasogastric tube feeding, swallowing training, rehabilitation exercises.

### 9b. Administration

• ✅ Phototherapy 12 hours daily, nasogastric feeding 80 ml every 3 hours, rehabilitation 5 days per week.

### 9c. Changes in intervention

• ✅ Transition from nasogastric to oral feeding after 10 rehabilitation sessions.

## 10. Follow-up and Outcomes

### 10a. Outcomes

• ✅ Partial improvement in feeding ability, but continued hypotonia and developmental delay.

### 10b. Follow-up test results

• ✅ Inflammatory markers elevated during pneumonia admission, SARS-CoV-2 positive.

### 10c. Intervention adherence

• ✅ Family reported good adherence to rehabilitation program.

### 10d. Adverse events

• ✅ Pneumonia and SARS-CoV-2 infection at 42 days of age.

## 11. Discussion

### 11a. Strengths and limitations

• ✅ Strength: Comprehensive genetic testing confirmed novel mutation. Limitation: Small sample size, limited long-term follow-up.

### 11b. Relevant literature

• ✅ Cited 9 references including original PURA syndrome description and genotype-phenotype correlation studies.

### 11c. Scientific rationale

• ✅ The c.465C>G mutation introduces a premature termination codon, likely impairing PURA protein function and causing neurodevelopmental abnormalities.

### 11d. Take-away lessons

• ✅ Early genetic testing should be considered in neonates with hypotonia and feeding difficulties to identify rare genetic disorders like PURA syndrome.

## 12. Patient Perspective

• ✅ Parent reported, "We were relieved to have a diagnosis but worried about our child's future. The rehabilitation has helped with feeding, but we know there will be ongoing challenges."

## 13. Informed Consent

• ✅ Written informed consent obtained from parents for publication of this case report.
